# Tracking the fate of adoptively transferred myeloid-derived suppressor cells in the primary breast tumor microenvironment

**DOI:** 10.1371/journal.pone.0196040

**Published:** 2018-04-20

**Authors:** Jaclyn Sceneay, Christoph M. Griessinger, Sabrina H. L. Hoffmann, Shu Wen Wen, Christina S. F. Wong, Sophie Krumeich, Manfred Kneilling, Bernd J. Pichler, Andreas Möller

**Affiliations:** 1 Tumor Microenvironment Laboratory, QIMR Berghofer Medical Research Institute, Herston, Australia; 2 Werner Siemens Imaging Center, Department of Preclinical Imaging and Radiopharmacy, Eberhard Karls University Tübingen, Tübingen, Germany; 3 Department of Dermatology, Eberhard Karls University Tübingen, Tübingen, Germany; 4 School of Medicine, University of Queensland, Brisbane, Australia; Istituto Superiore di Sanità, ITALY

## Abstract

Myeloid-derived suppressor cells (MDSCs) are a heterogeneous population of immature myeloid progenitor cells that are expanded in cancer and act as potent suppressors of the anti-tumor immune response. MDSCs consist of two major subsets, namely monocytic (M-) MDSCs and granulocytic (G-) MDSCs that differ with respect to their phenotype, morphology and mechanisms of suppression. Here, we cultured bone marrow cells with IL-6 and GM-CSF *in vitro* to generate a population of bone marrow MDSCs (BM-MDSCs) similar to G-MDSCs from tumor-bearing mice in regards to phenotype, morphology and suppressive-function. Through fluorescent labeling of these BM-MDSCs and optical imaging, we could visualize the recruitment and localization of BM-MDSCs in breast tumor-bearing mice *in vivo*. Furthermore, we were able to demonstrate that BM-MDSCs home to primary and metastatic breast tumors, but have no significant effect on tumor growth or progression. *Ex vivo* flow cytometry characterization of BM-MDSCs after adoptive transfer demonstrated both organ-and tumor-specific effects on their phenotype and differentiation, demonstrating the importance of the local microenvironment on MDSC fate and function. In this study, we have developed a method to generate, visualize and detect BM-MDSCs *in vivo* and *ex vivo* through optical imaging and flow cytometry, in order to understand the organ-specific changes rendered to MDSCs in breast cancer.

## Introduction

Expansion of suppressive cell populations represents a key strategy employed by tumors to escape immune surveillance. Myeloid-derived suppressor cells (MDSCs) are one such population, consisting of myeloid progenitor cells and immature myeloid cells [1,2]. This heterogeneous population can differentiate into mature granulocytes, macrophages and dendritic cells (DCs) in healthy individuals, but in cancer patients, inhibition of differentiation results in the accumulation of an immature population consisting of myeloid precursors [1,3]. The importance of immunosuppressive MDSCs in cancer is well established, with the number of circulating MDSCs correlating with metastatic burden and cancer stage [4]. However, their biology is not well understood. MDSCs were initially defined based on the co-expression of CD11b and Gr-1 in tumor-bearing mice [5,6]. More recently, two distinct Ly6 superfamily receptors, Ly6C and Ly6G, have been used to further define different MDSC subpopulations into CD11b^+^/Ly6C^med/int/+^/Ly6G^+^ polymorphonuclear or granulocytic MDSCs (G-MDSCs), and CD11b^+^/Ly6C^high/+^/Ly6G^-^ monocytic MDSCs (M-MDSCs) [1,7,8]. In humans, these granulocytic and monocytic populations are defined as CD11b^+^/CD14^-^/CD15^+^ and CD11b^+^/CD14^+^, respectively [2,9].

The immunosuppressive mechanisms of these subsets are also distinct. G-MDSCs suppress CD8^+^ T cells primarily through ROS production and arginase (Arg)-1, while M-MDSCs suppress CD8^+^ T cells via inducible nitric oxide synthase (iNOS) and reactive nitrogen species [3,10–12]. While G-MDSCs are the predominant population in tumor-bearing mice [3], the prevalence and ratio of the different MDSC subsets is highly variable depending on the tumor type and tissue in question [13], and much remains unknown regarding the factors that regulate their function and mobilization in different tumor models. G-MDSCs are generally the prevailing population in lymphoid organs, but this ratio can be skewed to favour M-MDSCs in the tumor, indicating that MDSC differentiation, migration and function is strongly influenced by factors in the local environment [14]. Cytokines, chemokines and pro-inflammatory factors produced by both tumor and stromal cells promote myelopoiesis, block maturation of myeloid cells and recruit MDSCs to the tumor microenvironment (TME) [2,9,15–24].

Suppressive cell populations such as MDSCs represent major impediments to the development of effective cancer treatments, as their ability to suppress anti-cancer immunity compromises the effectiveness of immune-based therapies [2]. Research efforts have focused on eliminating or inactivating MDSCs in order to restore anti-tumor immunity and reverse the suppressive state of the TME [25,26]. In order to successfully target MDSCs however, it is necessary to more closely understand the mechanisms governing their mobilization, trafficking, function and differentiation potential. As MDSCs do not exist in the same state in naïve mice [27], current methods of study involve enrichment and isolation through flow cytometry from the different tissues of tumor-bearing mice or patient samples, which can result in very low yields. MDSCs isolated from tumor-bearing mice do not survive long or proliferate in culture [28], and very few studies have investigated the potential to generate MDSCs *de novo*. Recently, a protocol to generate immunosuppressive MDSCs from bone marrow-derived cells (BMDCs) cultured with granulocyte macrophage colony stimulating factor (GM-CSF) and interleukin 6 (IL-6) was established [29]. GM-CSF has been identified as the main cytokine responsible for MDSC expansion *in vivo* in numerous studies [11,21,28,30–32], while the pro-inflammatory cytokine IL-6 reportedly contributes to MDSC accumulation and suppressive function [17,19,27]. Here, we utilize MDSCs generated from BMDCs *in vitro* with GM-CSF and IL-6 (referred to as BM-MDSCs) to visualize their recruitment and fate after adoptive transfer in breast tumor-bearing mice using optical imaging (OI). OI provides only 2D planar acquisitions with low penetration depths and distinct limitations. However, with near infrared dyes such as DiD it is possible to achieve a high sensitivity and increase the penetration depth, making it a powerful non-invasive imaging modality for cell tracking analyses [33]. In this study, we show that BM-MDSCs can be stably labeled with DiD and tracked *in vivo* by OI, and re-localized and characterized *ex vivo* by flow cytometry, allowing investigation of the localization and fate of DiD-labeled BM-MDSCs in breast tumor-bearing mice, as a novel method for identifying the organ-specific changes rendered to MDSCs in breast cancer.

## Materials and methods

### Mice

C57Bl/6 mice were purchased from the Walter and Eliza Hall Institute (Melbourne, Australia) or Charles River Laboratories (Sulzfeld, Germany) with female mice used at 8–14 weeks of age. Transgenic polyoma middle-T mouse mammary tumor virus (PyMT-MMTV) mice were generated as described [34]. OT-I mice [35] were a kind gift from the Smyth laboratory (QIMR Berghofer Medical Research Institute, Australia). All animal procedures were conducted in accordance with Australian and German National Health and Medical Research regulations on the use and care of experimental animals, and approved by the QIMR Berghofer Animal Ethics Committee (A1212-617N) and the Regierungspräsidium Tübingen, respectively. Animal condition (appearance, weight loss, ruffled fur, reduced mobility and body posture) was monitored every second day throughout all experiments to ensure animal well-being and used as humane endpoints if required. Mice were sacrificed by a chemical method (overdose of a barbiturate anesthetic) with a secondary physical method (cervical dislocation). No animals were euthanized prematurely in this study and all mice met criteria for euthanasia based on experimental endpoint.

### Cell lines

The wildtype PyMT(-WT) and luciferase positive (Luc)-PyMT mammary tumor cell lines were established, derived and maintained as previously described [34,36]. The lines were authenticated as mouse lines by STR profiling (conducted every 6 months by QIMR Berghofer core facility, last test results 7 December 2016) and confirmed to be mycoplasma free (conducted every 4 months and when cells taken from storage by QIMR Berghofer core facility, last test results 7 December 2016).

### BM-MDSC generation

The generation of *in vitro* bone marrow-derived MDSCs (BM-MDSCs) was adapted from Marigo et al [29]. BMDCs were flushed from the femur and tibia of mice and cultured for 5 days in ultra-low attachment petri dishes (Corning) with complete RPMI medium supplemented with β_2_-mercaptoethanol and 40 ng/ml GM-CSF and IL-6 (Peprotech) on day 1, 3 and 5. On day 6, BMDCs positive for CD11b, Ly6C and Ly6G (classified as BM-MDSCs) were obtained by Fluorescence Activated Cell Sorting (FACS) as described below and used for subsequent experiments.

### Cell labeling and viability

Sorted BM-MDSCs were labeled with Vybrant® DiD cell-labeling solution (Life Technologies) according to manufacturer’s instructions.

### Antigen-specific T cell proliferation assay

CD8^+^ T cells were isolated by FACS from the spleen and lymph nodes of OT-I mice and labeled with Carboxyfluorescein Diacetate Succinimidyl Ester (CFSE; Sigma) according to the manufacturer’s instructions. For co-culture, 1x10^5^ CFSE-labeled CD8^+^ T cells were cultured with 1x10^5^ irradiated splenocytes (50 Gy for 30 minutes) from naïve C57Bl/6 mice pulsed with SIINFEKL peptide (f.c.1 μM; Sigma) for 2 hours at room temperature. SIINFEKL is a MHC class I-restricted peptide epitope of ovalbumin (OVA) that can be used to detect an OVA-specific CD8+ cytolytic T cell response in cells derived from OT-I mice. BM-MDSCs (generated as above) were added at various ratios as indicated and incubated for 72 hours at 37°C. Proliferation of CD8^+^ T cells was assessed by flow cytometry as described below.

### Real-time PCR

*In vitro* cultured BM-MDSCs and G-MDSCs (CD11b^+^/Ly6C^med^/Ly6G^+^) isolated from the tumor, lung and spleen of PyMT-WT breast cancer-bearing and naive C57Bl/6 mice were homogenized in lysis buffer (peqGOLD Total RNA Kit, Peqlab) directly after magnetic cell sorting with the G-MDSC-kit (Miltenyi). Genomic DNA was digested during RNA preparation (PeqGOLD DNase I digest kit, Peqlab) and 1 μg of RNA was transcribed into cDNA (Superscript II Reverse Transcriptase; Invitrogen). Real-time PCR was performed in a reaction volume of 10 μL with 20 ng cDNA, 5 pmol of the respective primers and a SYBR Green master mix preparation (QuantiFast SYBR Green PCR Kit, Qiagen) using a LightCycler Real-Time PCR system (Roche Diagnostics) with the following cycling conditions: 95°C, 15 min; 95°C, 15 sec; 62°C, 45 sec; 72°C, 30 sec. For mRNA expression level analysis, all investigated genes were normalized to the housekeeping gene aldolase and CD11b/Ly6C^med^/Ly6G^+^ cells from the spleens of naïve mice using the ΔΔCT method. Primer sequences listed in S1 Table.

### Tumor models

For primary tumor growth, mice were orthotopically injected with 5x10^5^ PyMT-WT or Luc-PyMT mammary tumor cells resuspended in 20 μl PBS into the 4^th^ left mammary fat pad (MFP) under anesthetic. Tumor growth was monitored every 2–3 days using digital calipers. For survival experiments, mice were euthanized at experimental endstage defined as a total tumor volume of 525 mm^3^ (calculated as π x length x width^2^/6).

To assess metastatic tumor growth, 2.5x10^5^ Luc-PyMT cells resuspended in 100 uL PBS were injected intracardiacally under anesthetic. Metastatic tumor growth was monitored by OI imaging for bioluminescent signals. Mice were euthanized at experimental endpoint (5 weeks after tumor cell injection).

### BM-MDSC adoptive transfer

For adoptive transfer of BM-MDSCs, 1x10^6^ or 4x10^6^ DiD-labeled or unlabeled BM-MDSCs were injected in 100 μl PBS into mice via intravenous (i.v.; tail vein) in C57Bl/6 mice bearing PyMT-WT or Luc-PyMT primary and metastatic tumors at indicated timepoints, or via intraperitoneal (i.p.) or intracardiac (i.c.) injections in naive C57Bl/6 mice.

### Optical imaging

Mice were injected with 150 mg/kg Luciferin (Perkin Elmer) i.p. for bioluminescence (BL) scans. After a 2 minute uptake period, fluorescence (FL; to visualize BM-MDSCs) and BL (to visualize tumor burden) scans were carried out on an Ivis Spectrum OI system (Perkin Elmer). BL scans were acquired for 20, 60 and 120 seconds and signals were normalized for the photon radiance (photons/second/cm^2^/sr). DiD-FL scans were acquired for 1 and 2 seconds with a 640nm excitation and 680nm emission filter and normalized for the radiant efficiency [(photons/second/cm^2^/sr)/mW x cm^2^]. OI scans were carried out at timepoints indicated and were analyzed using Living Image Software 4 (Perkin Elmer). Regions of interest were drawn on target organs and background areas in *ex vivo* OI scans to acquire mean values for the calculation of organ/background-ratios.

### Flow cytometry

Tumors and lungs were cut into small pieces and digested with 0.2 mg/mL collagenase type IV (Worthington Biochemical Corp.) and 0.02 mg/mL DNase I (Roche) at 37°C for 45 min and 30 min respectively. Single cell suspensions of lung, spleen, tumor or BM-MDSCs were stained with relevant anti-mouse antibodies in the presence of anti-CD16/32 to block Fc-receptors on ice. Non-viable cells were excluded on the basis of staining with 7-aminoactinomycin or Zombie Yellow (BioLegend). Flow cytometry acquisition and sorting were carried out on a FACS Fortessa 4 and Aria II (BD Biosciences) respectively. Data analysis was performed using FlowJo software (Tree Star). Antibody details listed in S2 Table.

### Cytospin

Myeloid cells isolated by FACS from the tumor, lung and spleen were resuspended at 2x10^5^ cells/mL in PBS and spun onto slides using a Cytospin 3 centrifuge (Shandon) at 500 rpm for 5 minutes. Slides were Diff-Quick stained the next day and images taken using a Leica DMIRB microscope with NIS Elements software.

### Statistical analyses

Results are expressed as mean ± SEM and analyzed by one-way ANOVA with Tukey’s multiple comparison test for significance. Kaplan-Meier Survival curves were analyzed using Log-rank (Mantel-Cox) Tests; p values <0.05 were considered significant (****p<0.0001, ***p<0.001, **p<0.01 and *p<0.05).

## Results

### BM-MDSCs generated *in vitro* share similar properties with G-MDSCs from tumor-bearing mice

**Fig 1:**
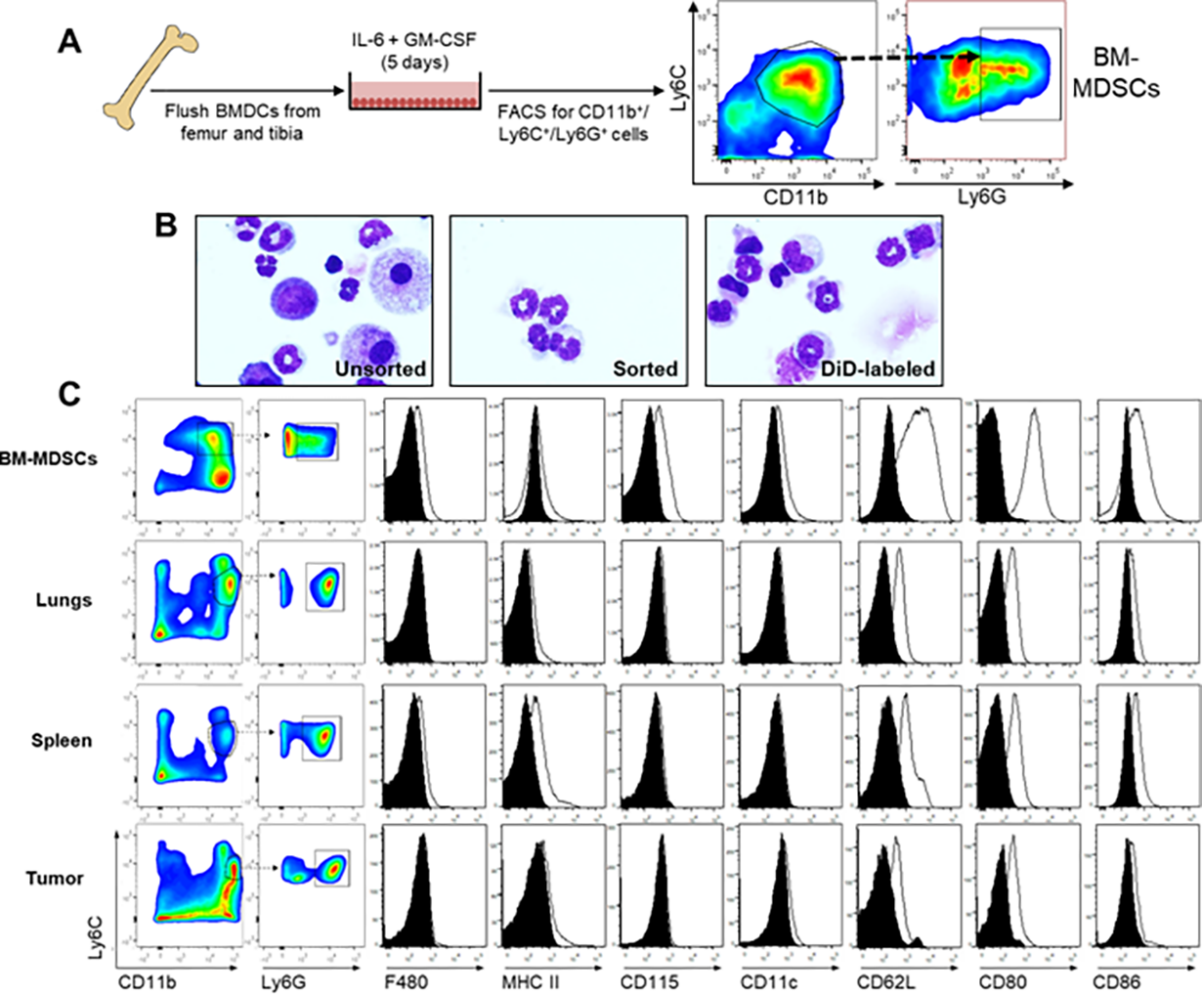
Characterization of *in vitro*-cultured BM-MDSCs. **A**) Schematic of *in vitro* BM-MDSC generation. BMDCs flushed from the femur and tibia of naïve C57Bl/6 mice were cultured with IL-6 and GM-CSF for 5 days and isolated by FACS based on CD11b^+^/Ly6C^+^/Ly6G^+^ expression. **B**) Representative Diff-Quick stained cytospin images of CD11b^+^/Ly6C^+^/Ly6G^+^ cells. Images show *in vitro*-cultured BMDCs before FACS (left; unsorted), BM-MDSCs after FACS (middle; sorted) and DiD-labeling (right). Images were taken at 60x magnification. **C**) Gating strategy and characterization of *in vitro* cultured CD11b^+^/Ly6C^+^/Ly6G^+^ BM-MDSCs (top row) and CD11b^+^/Ly6C^med^/Ly6G^+^ G-MDSCs isolated by FACS from the lung (second row), spleen (third row) and tumor (bottom row) of PyMT-MMTV transgenic mice, for the expression of various surface markers by flow cytometry.

**Fig 2:**
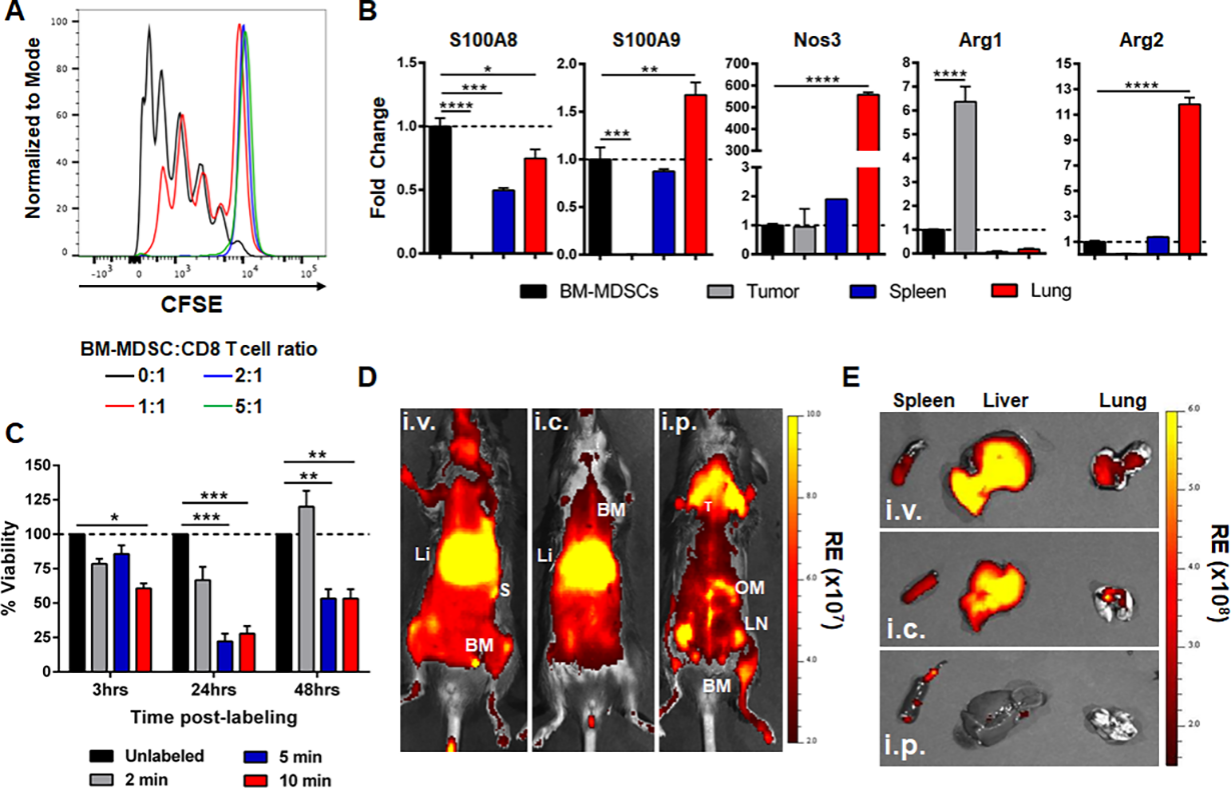
*In vitro* function and localization of BM-MDSCs *in vivo*. **A**) Representative histogram of CFSE-labeled CD8 OT-I T cells co-cultured with splenocytes and BM-MDSCs at various ratios as indicated (n = 3 in triplicate). **B**) RT-PCR with fold change gene expression in G-MDSCs isolated from the tumor, spleen and lungs of PyMT-WT tumor-bearing mice normalized to gene expression in BM-MDSCs (indicated by broken line; n = 3 in triplicate). **C**) Percent viability of BM-MDSCs at 3, 24 and 48 hours after initial DiD-labeling for 2, 5 or 10 min relative to unlabeled cells (broken line). Data represented as mean ± SEM. *p<0.05; **p<0.01; ***p<0.001. **D, E**) OI *in vivo* (**D**) and of *ex vivo* organs (**E**) 7 days after i.v., i.c. or i.p. injection of 1x10^6^ DiD-labeled BM-MDSCs into naïve mice. BM = bone marrow, Li = liver, LN = lymph nodes, OM = omentum majus, S = spleen, T = thymus). RE = Radiant Efficiency; R = Radiance.

Treatment of BMDCs with GM-CSF and IL-6 (Fig 1A) resulted in the expansion of a population of myeloid cells sharing similarities with MDSCs as described [29]. We decided to focus specifically on the G-MDSC-like population of BM-MDSCs, as this is the predominant population in tumor-bearing mice and humans [3]. Furthermore, previous work from our lab had shown that CD11b^+^/Ly6C^med^/Ly6G^+^ G-MDSCs were important in creating an immune-suppressed pre-metastatic niche in breast cancer [36]. Therefore, we isolated the CD11b^+^/Ly6C^+^/Ly6G^+^ fraction (BM-MDSCs) by flow cytometry (Fig 1A) for all further experiments. This fraction demonstrated the distinct multi-lobed nuclear morphology associated with G-MDSCs, compared to the mixed population of myeloid precursors and blast cells present before sorting (Fig 1B). In order to determine how closely these BM-MDSCs resembled their counterparts in tumor-bearing mice, we analyzed G-MDSCs from the spleen, lungs and tumors of PyMT-MMTV mice that develop tumors spontaneously in the mammary fat pad, for the expression of various cell surface receptors, and compared this with BM-MDSCs generated with GM-CSF and IL-6 *in vitro* (Fig 1C). While BM-MDSCs did not demonstrate the distinct intermediate and high Ly6C^+^ populations present in other organs, the CD11b^+^/Ly6C^+^/Ly6G^+^ fraction showed similarly low levels of F4/80, MHC Class II and CD11c, and increased CD115 expression (Fig 1C). All G-MDSCs expressed CD62L, CD80 and CD86 in varying degrees, with BM-MDSCs demonstrating the highest expression of these receptors. We next wanted to characterize the suppressive functions of these *in vitro* generated-BM-MDSCs. In order to do this, we first assessed the ability of BM-MDSCs to suppress proliferation of antigen-specific CD8^+^ T cells, by co-culturing BM-MDSCs at various ratios with CFSE-labeled CD8^+^ T cells isolated from OT-I mice, and SIINFEKL peptide-pulsed irradiated splenocytes. We found that BM-MDSCs could partially suppress T cell proliferation at a 1:1 ratio of BM-MDSCs to T cells, and completely at a 2:1 and 5:1 ratio (Fig 2A). We also assessed gene expression of various factors associated with the suppressive function of MDSCs including S100A8, S100A9, Nos3, Arg1 and Arg2 in BM-MDSCs compared to G-MDSCs from the tumor, spleen and lungs of tumor-bearing mice (Fig 2B). We found that BM-MDSCs expressed similar levels of S100A9, Nos3 and Arg2 compared to splenic G-MDSCs (Fig 2B), but that there were notable organ-specific differences in gene expression. Together, this data demonstrates that CD11b^+^/Ly6C^+^/Ly6G^+^ BM-MDSCs generated using IL-6 and GM-CSF *in vitro*, display similar morphological and functional properties to G-MDSCs expanded in the presence of a mammary tumor *in vivo*.

### DiD-labeled BM-MDSCs home to primary breast tumors *in vivo*

We next examined the dynamics of these BM-MDSCs in breast cancer, particularly in regards to their localization and homing to different sites after adoptive transfer. In order to visualize BM-MDSCs *in vivo*, we labeled them with DiD, a near-infrared fluorescent dye ideal for OI studies. We found that DiD-labeling of BM-MDSCs for 2 minutes did not significantly affect the viability of the cells after 48 hours, while longer incubation times impacted viability over time (Fig 2C). By using different routes of administration (i.v., i.c., or i.p.) in naïve C57Bl/6 mice, we were able to visualize the accumulation of adoptively transferred DiD-BM-MDSCs (1x10^6^ cells) in different organs (Fig 2D and 2E and S1 Fig). Intravenous and intracardiac injection led to an accumulation in the lung, liver, spleen and bone marrow (sternum, femur) after 7 days. After i.p. injection, DiD-BM-MDSCs were detected mainly in lymphoid tissues attached to the spleen (omentum majus, minor), lymph nodes and bone marrow (sternum, femur). We chose an i.v. route for adoptive transfer of BM-MDSCs for all further experiments, as this route of administration resulted in the accumulation of DiD-BM-MDSCs in sites most commonly affected by metastasis in breast cancer patients, including the bone, lung and liver.

**Fig 3:**
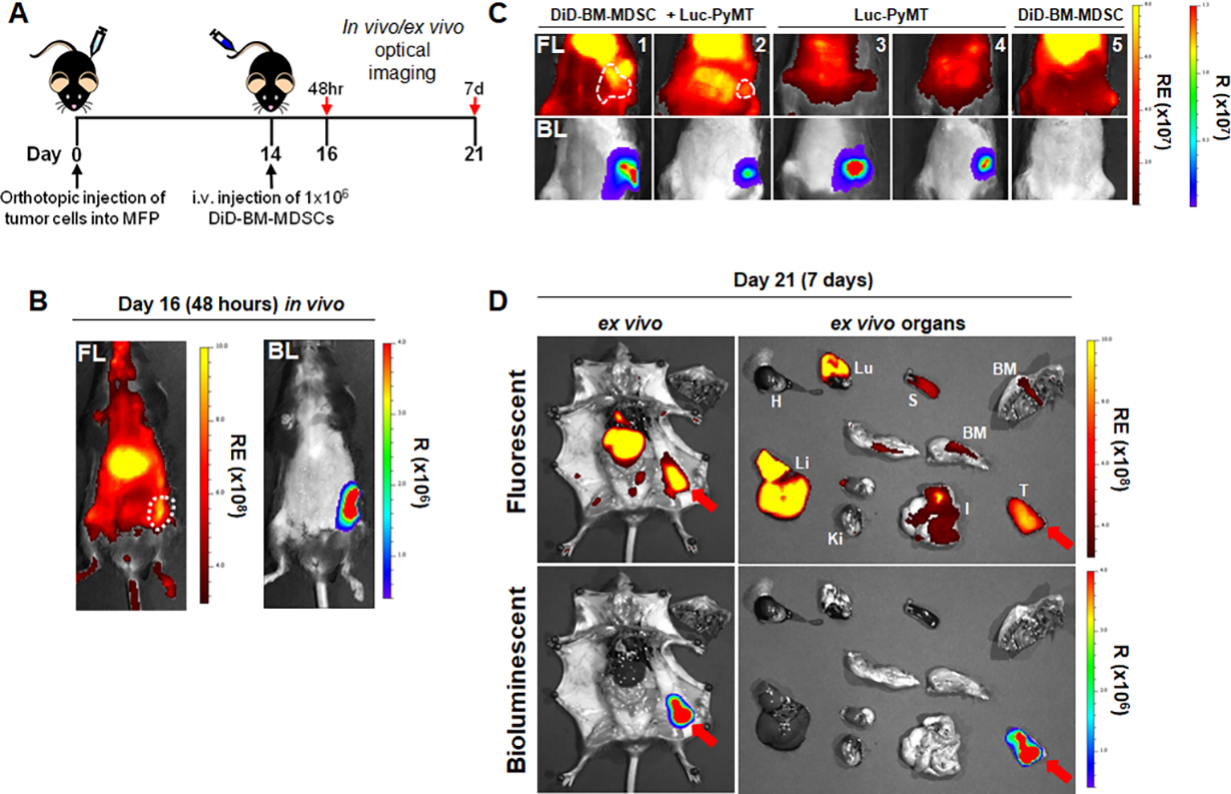
DiD-BM-MDSCs home to the primary breast tumor after adoptive transfer. **A**) Schematic of treatment regimen for localization of DiD-BM-MDSCs in tumor-bearing mice. Mice were injected with 2.5x10^5^ Luc-PyMT cells into the MFP on day 0, and i.v. injected with 1x10^6^ DiD-BM-MDSCs on day 14. *In vivo* optical images were obtained at 48 hours (day 16) and 7 days (day 21) post-injection of DiD-BM-MDSCs. *Ex vivo* images were taken at 7 days post-injection (day 21). **B**) Representative *in vivo* FL and BL optical images on day 16; 48 hours after injection of DiD-BM-MDSCs. Tumors outlined by broken white line. **C)** Representative *in vivo* FL and BL optical images of mice from (A) on day 21; 7 days after injection of DiD-BM-MDSCs. Treatment groups include mice with Luc-PyMT tumors and adoptively transferred DiD-BM-MDSCs (mice 1–2; left), Luc-PyMT tumors alone (mice 3–4; middle) or DiD-BM-MDSCs alone (mouse 5; right). Tumors outlined by broken white line. **D**) Representative *ex vivo* FL and BL optical images of Luc-PyMT tumor-bearing mice (left) and individual organs (right) on day 21; 7 days after DiD-BM-MDSCs injection. Tumor indicated by red arrow. BM = bone marrow, H = heart, I = intestine, Ki = kidney, Li = liver, LN = lymph nodes, Lu = lungs, S = spleen, T = tumor. RE = Radiant Efficiency; R = Radiance.

Next, we examined if adoptively transferred DiD-BM-MDSCs would additionally home to the primary tumor site in mice. We used Luc-PyMT breast tumor cells to contrast the BL signal from the primary tumor with the FL signal from the DiD-BM-MDSCs. Two weeks after Luc-PyMT cells were injected into the MFP, DiD-BM-MDSCs were adoptively transferred (i.v.) into these mice and OI carried out 48 hours and 7 days later (Fig 3A). Within 48 hours after DiD-BM-MDSC administration (day 16), a FL signal was detected which overlayed with the BL signal from the primary tumor (Fig 3B), indicating homing of DiD-BM-MDSCs to the tumor. This co-localization was still detectable *in vivo* 7 days after adoptive transfer (Fig 3C). *Ex vivo* OI (Fig 3D and S1 Fig) confirmed homing of DiD-BM-MDSCs to various organs at day 7 after adoptive transfer as in Fig 2D and 2E; with the strongest signals detected in the tumor, lung and liver. This data demonstrates that BM-MDSCs home to the primary tumor and accumulate in various organs after adoptive transfer.

### DiD-labeled BM-MDSCs home to established metastatic tumors *in vivo*

**Fig 4:**
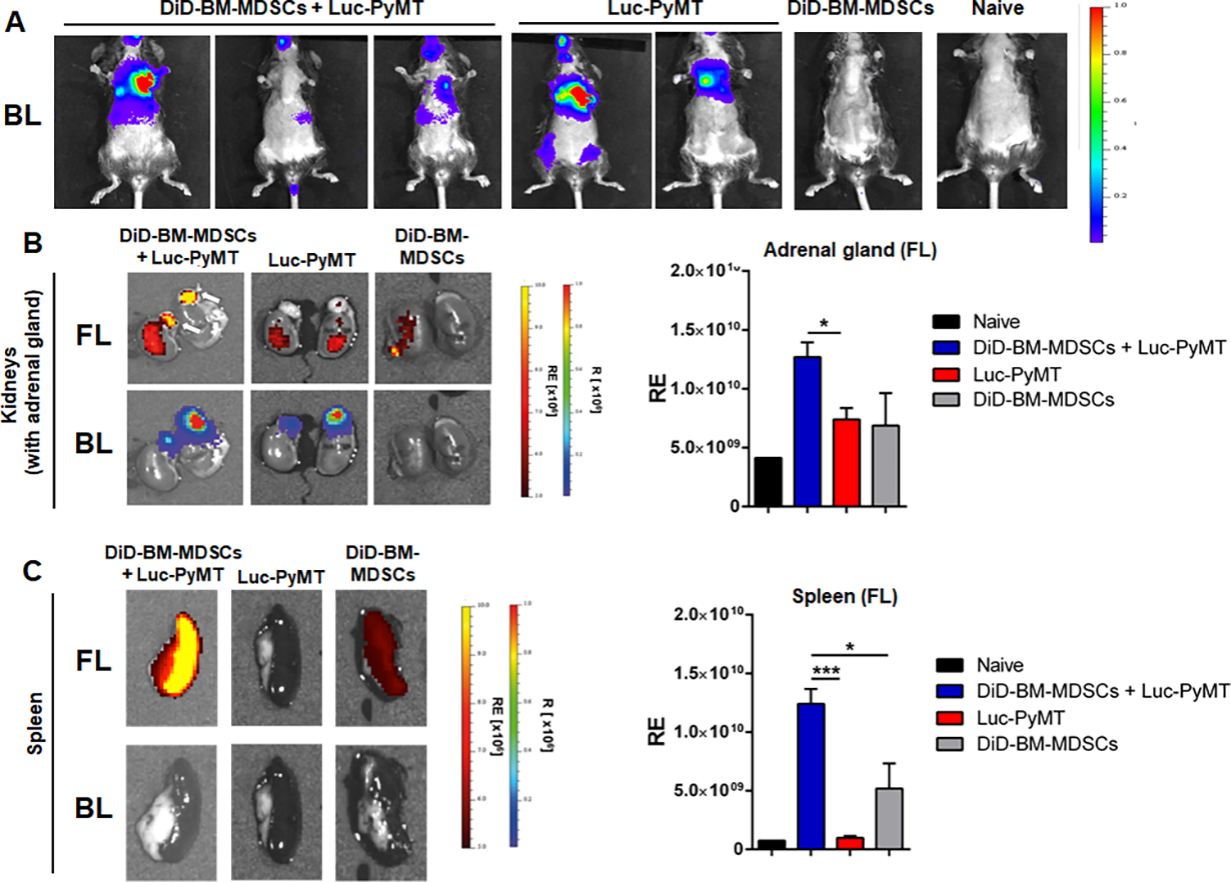
Homing of adoptively transferred DiD-BM-MDSCs to established metastases. **A**) Representative *in vivo* images of Luc-PyMT metastases (BL signal) 3 weeks after i.c. injection into C57Bl/6 mice. **B**) Representative e*x vivo* images of DiD-BM-MDSC (FL signal; top panel) localization to adrenal gland metastases (BL signal; bottom panel). DiD-BM-MDSCs were injected (i.v.) into mice from A, and images acquired 2 weeks later. Quantification of radiant efficiency (RE) of FL-signal for the adrenal gland shown on the right. **C**) Representative images of spleens (left) and RE quantification (right) from treatment groups described in **A and B**. Naïve C57Bl/6 mice were used as controls. Data represented as mean ± SEM; *p<0.05; ***p<0.001; n = 3–5 mice for all groups.

Given that adoptively transferred BM-MDSCs could home to primary breast tumors, we next wanted to determine whether the same would also occur in the context of established metastatic lesions. As spontaneous metastasis rarely occurs after injection of PyMT-WT cells in the MFP in this model, we injected Luc-PyMT cells i.c. and three weeks later, once metastatic tumors were confirmed to be present by OI (Fig 4A), DiD-BM-MDSCs were adoptively transferred (i.v.) into these mice. In *ex vivo* OI measurements, we could track the DiD-BM-MDSC signal to adrenal gland metastases 2 weeks after adoptive transfer (Fig 4B). FL-signals of DiD-BM-MDSCs (1.27x10^10^±1.25x10^9^ Radiant Efficiency, RE) in adrenal gland metastases were increased 1.8-fold over FL-signals of control animals (DiD-BM-MDSCs alone: 6.88x10^9^±2.76x10^9^ RE; Luc-PyMT alone: 7.39x10^9^±9.83x10^8^ RE). We could also observe a 2.4-fold increase in the DiD-BM-MDSC signal in the spleens of metastases-bearing animals (1.24x10^10^±1.27x10^9^ RE) compared to animals receiving DiD-BM-MDSCs alone (5.19x10^9^±2.15x10^9^ RE; Fig 4C) but not in the lungs or liver (S2 Fig). This suggests preferential localization of BM-MDSCs to the spleen in tumor-bearing compared to tumor-free animals, even though no BL signal was detected in the spleen (Fig 4C). Together, this data demonstrates that adoptively transferred DiD-BM-MDSCs can home to the sites of primary and metastatic tumors, and additionally accumulate in the spleens of tumor-bearing animals.

### Adoptive transfer of DiD-BM-MDSCs does not alter primary breast tumor growth

As these BM-MDSCs were shown to demonstrate suppressive properties (Fig 2A and 2B), we next wanted to investigate whether the accumulation of BM-MDSCs in the primary tumor impacted growth. We examined this in two different ways. First, mice bearing PyMT-WT tumors were i.v. injected with two doses of either 1x10^6^ or 4x10^6^ BM-MDSCs at day 10 and 20 after tumor cell injection, and primary tumor growth monitored to endstage (S3 Fig). In both cases, adoptive transfer of BM-MDSCs into tumor-bearing mice did not impact tumor growth or overall survival compared to control tumor-bearing mice (S3 Fig). Therefore, although we found that adoptively transferred BM-MDSCs home to the primary tumor site (Fig 2), these BM-MDSCs did not affect their growth.

Secondly, we examined whether the presence of BM-MDSCs at the initiation of tumor development would affect the growth kinetics of the primary tumor. To do this, BM-MDSCs were co-injected with PyMT-WT tumor cells at a ratio of 1:1 into the mammary fat pad and tumor growth monitored. However, no differences in tumor growth or overall survival were observed compared to mice injected with PyMT-WT cells alone (S4 Fig). Therefore although BM-MDSCs were able to suppress T cell function *in vitro*, this did not translate into an effect on tumor growth *in vivo*. This data is in accordance with previously published studies, demonstrating that CD11b^+^/Gr1^high^ MDSCs are the least suppressive subpopulation of MDSCs and do not inhibit breast tumor growth after adoptive transfer *in vivo* [11].

### Changes in the breast tumor microenvironment after BM-MDSC adoptive transfer

As DiD-BM-MDSCs were shown to home to but not affect the growth of primary breast tumors, we next wanted to analyze the composition of the TME relative to control mice. Similar to the homing experiments in Fig 3, mice were injected with 1x10^6^ DiD-BM-MDSCs on days 10 and 20 after PyMT-WT tumor cell injection into the MFP (S5 Fig). Mice were sacrificed 48 hours (day 22), 5 days (day 25) or 7 days (day 27) after the second DiD-BM-MDSC injection and tumors, lungs and spleen harvested and analyzed by flow cytometry for different stromal populations.

Within the TME, we observed a significant decrease in the overall frequency of CD3^+^ lymphocytes at 48 hours and 7 days after DiD-BM-MDSC injection compared to tumor-bearing controls (S5 Fig). After 48 hours, we also observed a significant increase in the frequency of Ly6C^+^ cells within the CD11b^+^ myeloid population in the primary tumor (S5 Fig); however this was not detectable at day 7. When we focused on the MHC II^+^/CD45.2^+^ myeloid populations in particular, we found that generally, the frequency of macrophages (CD11b^+^/F480^+^) and DCs (CD11c^+^) was increased after 48 hours compared to controls, but that these differences were not apparent at day 7 (S5 Fig). Therefore, adoptive transfer of BM-MDSCs appeared to alter the TME within 48 hours after injection, however, most of these changes were undetectable by day 7.

We also analyzed the same populations in the spleen and lungs of these mice at 48 hours and 5 days after injection. Interestingly, the dynamics of the cell populations appeared to be organ-dependent. In the spleen at 48 hours, no changes in lymphocytes were observed, but Ly6C^+^ and F480^+^ populations of CD11b^+^ cells were decreased (S6 Fig). However, most of these changes were not apparent after 5 days. In the lungs, we did not observe any significant changes to any cell populations after 48 hours or 5 days (S6 Fig).

### The fate of adoptively transferred DiD-BM-MDSCs is dictated by the local environment

While accumulation of adoptively transferred BM-MDSCs might be expected to alter the local organ environment as described above, similarly, the local environment could also impact BM-MDSCs. Given the importance of tumor-derived factors on MDSC mobilization and function, we reasoned that microenvironmental influences may either maintain the BM-MDSCs in an immature state or promote their differentiation into mature myeloid populations. Therefore, in order to track the fate of these BM-MDSCs in specific organs after adoptive transfer, we utilized the DiD fluorescence signal to examine BM-MDSCs *ex vivo* by flow cytometry.

**Fig 5:**
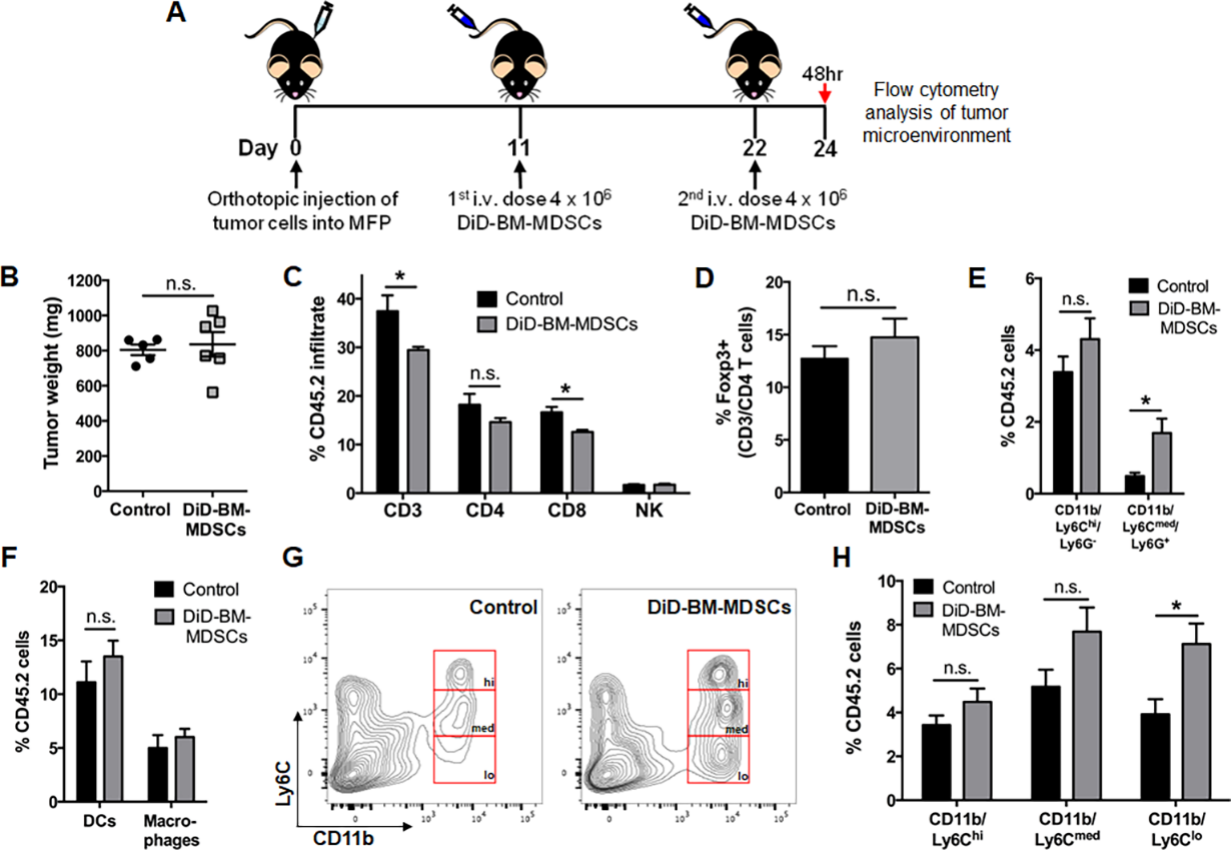
Breast tumor microenvironment dynamics after adoptive transfer of DiD-BM-MDSCs. **A**) Schematic of treatment regimen for TME analysis after adoptive transfer of DiD-BM-MDSCs into tumor-bearing mice. Mice were injected with PyMT-WT cells into the MFP on day 0, and i.v. injected with 4x10^6^ DiD-BM-MDSCs on day 11 and 22. Tumors were analyzed by flow cytometry 48 hours (day 24) after the second dose of DiD-BM-MDSCs. PyMT-WT tumor-bearing mice were used as controls (control n = 5; DiD-BM-MDSC n = 6 for all analyses). **B**) Tumor weight at time of flow cytometry analysis at day 24. **C**) Flow cytometry analysis of CD3 lymphocytes, CD3/CD4 and CD3/CD8 T cells, as well as CD3^-^/NK1.1^+^ NK cells within the tumor at day 24. **D**) Flow cytometry analysis of the percentage of Foxp3^+^ cells within the CD3/CD4 T cell population at day 24. **E**) Flow cytometry analysis of CD11b/Ly6C/Ly6G myeloid populations as a percentage of CD45.2^+^ cells within the tumor at day 24. **F**) Flow cytometry analysis of DCs (CD11c/MHC Class II) and macrophages (CD11b/F480/MHC Class II) as a percentage of CD45.2^+^ cells within the tumor at day 24. **G**) Representative flow cytometry plots of CD11b/Ly6C populations (hi = high, med = medium and lo = low) within the CD45.2^+^ population in the tumor at day 24 and quantified in **H.** Data represented as mean ± SEM. *p<0.05; n.s. not significant.

From the above experiments, we were able to conclude that the most significant changes to local organ environments were apparent within 48 hours after adoptive transfer of BM-MDSCs. Furthermore, we were confident that these cells remained viable at this time point based on our *in vitro* viability studies (Fig 2). Therefore, in order to better visualize and analyze the fate of DiD-BM-MDSCs, we adoptively transferred two doses of 4x10^6^ DiD-BM-MDSCs (total of 8x10^6^ cells) and conducted flow cytometry analysis at the 48-hour timepoint (Fig 5A). DiD-BM-MDSC dosing was adjusted to days 11 and 22 and flow cytometry performed on day 24 to ensure similar primary tumor sizes (at BM-MDSC dosing) compared to previous experiments. Adoptive transfer of 8x10^6^ DiD-BM-MDSCs did not impact tumor growth or weight (Fig 5B), and given the lack of stromal cell changes previously observed in the lung, we focused on the primary tumor and spleen for further analysis.

Again when we analyzed the overall changes within the TME, CD3^+^ lymphocytes were decreased after adoptive transfer of DiD-BM-MDSCs compared to controls (Fig 5C), but this time specifically in regards to CD8^+^ T cells. CD4^+^ T cells, Natural Killer (NK) cells and Regulatory T cells (Tregs) were unchanged (Fig 5C and 5D). Of the general myeloid populations, only CD11b^+^/Ly6C^med^/Ly6G^+^ cells were increased (Fig 5E), while DCs and macrophages remained unchanged (Fig 5F). Within the CD11b^+^/Ly6C^+^ subset however, we observed a distinct CD11b^+^/Ly6C^lo^ population that was significantly increased in the primary tumor after DiD-BM-MDSC injection (Fig 5G and 5H). Interestingly, these changes were not observed in the myeloid cell populations in the spleens of these mice (S7 Fig).

**Fig 6:**
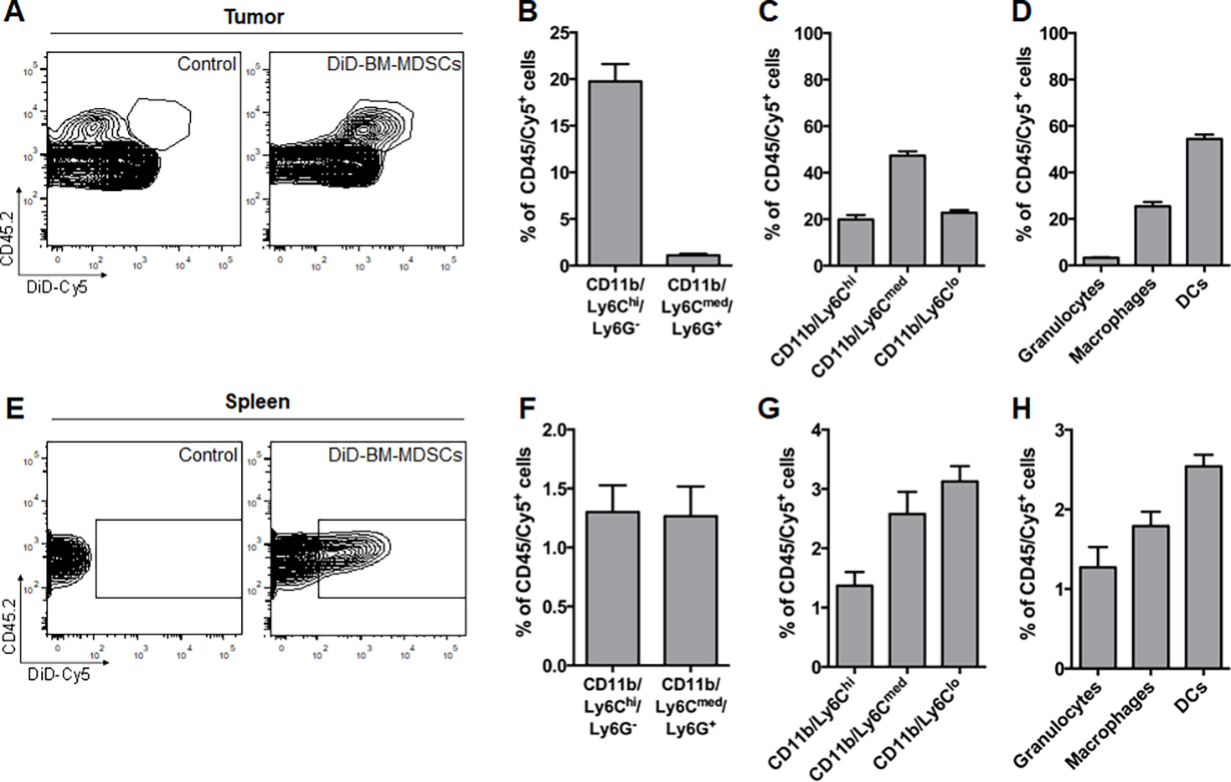
Fate of adoptively transferred DiD-BM-MDSC in the tumor and spleen of breast tumor-bearing mice. **A**) Representative flow cytometry plots of the CD45.2/DiD-Cy5^+^ gated population in PyMT-WT tumors from control mice (primary tumor alone; left) and after adoptive transfer of DiD-BM-MDSCs (right) on day 24 as in Fig 5**B and** 5**C**) Flow cytometry analysis of CD11b/Ly6C/Ly6G (B) and CD11b/Ly6C subpopulations (C) as a percentage of CD45/Cy5^+^ cells within the tumor. **D**) Flow cytometry analysis of mature myeloid populations including granulocytes (CD11b/Ly6G), macrophages and DCs as a percentage of CD45/Cy5^+^ cells within the tumor. **E**) Representative flow cytometry plots of the CD45.2/DiD-Cy5^+^ gated population within the spleen of PyMT-WT tumor-bearing mice (control; left) and after adoptive transfer of DiD-BM-MDSCs (right). **F,G**) Flow cytometry analysis of CD11b/Ly6C/Ly6G (F) and CD11b/Ly6C subpopulations (G) as a percentage of CD45/Cy5^+^ cells within the spleen. **H**) Flow cytometry analysis of mature myeloid populations including granulocytes, macrophages and DCs as a percentage of CD45/Cy5^+^ cells within the spleen. Data represented as mean ± SEM; n = 6 for all groups.

We then focused our analysis on the DiD^+^ population (detected as Cy5 by flow cytometry) of CD45^+^ cells (CD45^+^/Cy5^+^) at day 24 (Fig 6A) to determine the specific fate of the adoptively transferred DiD-BM-MDSCs. In the primary tumor, very few CD11b/Ly6C^med^/Ly6G^+^ cells (1.1±0.2%) could be detected relative to CD11b/Ly6C^hi^/Ly6G^-^ cells (19.7±1.9%) (Fig 6B). Within the Cy5^+^-Ly6C populations however, although Ly6C^med^ cells were the predominant population (47.3±4.4%), both Ly6C^hi^ (19.9±1.8%) and Ly6C^lo^ (22.8±1.1%) cells were also present (Fig 6C). Given the spread of Ly6C^hi^, Ly6C^med^ and Ly6C^lo^ cells observed, we also analyzed mature myeloid populations to determine if the adoptively transferred DiD-BM-MDSCs may have differentiated within the TME. Within the CD45/Cy5^+^-population, DCs made up more than half of the population (54.4±1.9%), followed by macrophages (25.4±1.8%) and granulocytes (3.3±0.4%) (Fig 6D), suggesting preferential differentiation of DiD-BM-MDSCs to DCs and macrophages within the tumor.

These changes were not observed in spleen however, with CD11b/Ly6C^med^/Ly6G^+^ and CD11b/Ly6C^hi^/Ly6G^-^ cells making up only a very small percentage of the overall CD45/Cy5^+^ population (1.2±0.3% and 1.3±0.2% respectively) (Fig 6E and 6F). This was also observed in the proportion of Ly6C^hi^ (1.3±0.2%), Ly6C^med^ (2.6±0.4%) and Ly6C^lo^ (3.1±0.3%) cells within the CD45/Cy5^+^ population (Fig 6G). DCs were again the most prominent of the mature myeloid populations (2.5±0.1%) compared to macrophages (1.8±0.2%) and granulocytes (1.3±0.3%) (Fig 6H). However the frequency of these mature myeloid populations within the Cy5^+^ population was negligible. Taken together, this data suggests that the local environment within particular organs play a crucial role in dictating the fate of adoptively transferred BM-MDSCs *in vivo*.

## Discussion

In this study, we show that BM-MDSCs generated *in vitro* using BMDCs treated with IL-6 and GM-CSF, show similar morphological and functional properties to G-MDSCs from breast tumor-bearing mice. Furthermore that fluorescent labeling of these BM-MDSCs allows us to track their homing and localization after adoptive transfer through OI, and determine their fate *ex vivo* through flow cytometry. Here we demonstrate, for the first time, that these adoptively transferred BM-MDSCs are actively recruited to tumor sites (primary or metastatic) where they are incorporated into the TME. After i.v. administration into naïve mice, DiD-BM-MDSCs were only shown to accumulate in the lung, liver and spleen. In tumor-bearing mice however, DiD-BM-MDSCs co-localized to the primary tumor and with metastatic tumors in the adrenal gland. This data is in accordance with previous studies showing that MDSCs actively migrate to tumor sites [37]. Although accumulation of BM-MDSCs did not affect primary tumor growth, even after adoptive transfer of 8x10^6^ cells, this finding is consistent with previous studies in which adoptively transferred G-MDSCs were found to be poorly immunosuppressive *in vivo* and failed to prevent tumor growth even in high numbers [11,29].

Accumulation of DiD-BM-MDSCs in tumor-bearing mice was not just limited to the sites of tumor growth. DiD-BM-MDSCs were also increased in the spleens of tumor-bearing mice compared to cancer-free controls. MDSCs display functional differences and varied mechanisms of suppression depending on their location at the primary tumor or peripheral sites [38,39]. Accordingly, we observed differences in expression of various genes associated with the suppressive function of MDSCs, based on tissue of origin. Additionally, we demonstrated that the adoptive transfer of DiD-BM-MDSCs into tumor-bearing mice induces distinct changes to the composition of the local microenvironment, as well as the fate of the DiD-BM-MDSCs in the spleen and tumor.

MDSCs rely on various tumor-derived cytokines and growth factors for their mobilization, expansion, polarization and immunosuppressive function (reviewed in [1]). Indeed recent studies indicate that the TME can directly alter the properties of MDSCs and subsequently their function. For example, GM-CSF within the TME has been shown to expand Gr1^int^ MDSCs (M-MDSC; Ly6C^hi^ equivalent), as well as a Gr1^lo^ (Ly6C^lo^ equivalent) fraction [11,32]. In this study, BM-MDSCs generated *in vitro* were morphologically similar to G-MDSCs (from tumor-bearing mice) and were able to suppress antigen-specific T cell proliferation, consistent with previous reports [29]. *Ex vivo* flow cytometry of these DiD-BM-MDSCs after adoptive transfer however, demonstrated that very few remained as CD11b/Ly6C^+^/Ly6G^+^ cells in the tumor, spleen or lung.

In the tumor, almost 20% of the DiD^+^ (Cy5^+^) population resembled M-MDSCs in regards to cell surface receptor expression (CD11b/Ly6C^hi^/Ly6G^-^), with Ly6G expression mostly absent. As only CD11b/Ly6C^+^/Ly6G^+^ BM-MDSCs with a polymorphonuclear morphology were adoptively transferred into mice, this suggests some of kind of plasticity within the M-MDSC and G-MDSC populations. Whether MDSCs can switch between the monocytic and granulocytic fractions is controversial, with G-MDSCs believed to be terminally differentiated [1]. However, Ly6C^hi^, Ly6C^med^ and a distinct Ly6C^lo^ subset were evident within the tumor-derived DiD-Cy5^+^ population 48 hours after adoptive transfer, suggesting differentiation of BM-MDSCs into other myeloid cell subsets. Furthermore, this apparent switch to M-MDSC-like cells within the TME is important, as G-MDSCs are thought to be terminally differentiated with only M-MDSCs reported to differentiate into macrophages and DCs [3]. The increased proportion of DCs and macrophages within the DiD-Cy5^+^ population might be reflective of differentiation of M-MDSCs within the TME. In support of this, M-MDSCs recruited to tumors have been demonstrated to rapidly differentiate into immunosuppressive macrophages and DCs (whose suppressive nature is still unknown) in the presence of tumor-derived soluble factors [2,38,40,41]. In our studies, whether the resulting DiD-Cy5^+^ macrophages and DCs are immunosuppressive remains to be investigated. However, recent work has shown that MDSCs can be reprogrammed by the TME to become tolerogenic DCs [42]. While further characterization of the DiD-Cy5^+^ DC population would need to be carried out, the distinct CD11b/Ly6C^lo^ population only evident in the tumor after DiD-BM-MDSC adoptive transfer suggests this may be an important avenue of investigation. Taken together, our data suggests that factors within the primary breast TME potentially promote differentiation of adoptively transferred BM-MDSCs into M-MDSCs, as well as macrophages and DCs.

In this study, we have also developed a tool that will allow us to address these additional questions. The *in vitro* generation of BM-MDSCs and ability to detect them through OI and flow cytometry once labeled with DiD, provide opportunities to interrogate this dynamic interaction of MDSCs within the TME as well as the local environments of other organs in the short-term. Although lipophilic dyes such as DiD are described to stably incorporate into the cell membrane and be transferred to daughter cells alone [43], contamination to neighboring cells has been reported [44]. We found that BM-MDSCs generated from the bone marrow of eGFP C57Bl/6 mice were not sufficiently detectable by flow cytometry within the tumor, due to high background signals and autofluorescence. Therefore in future studies requiring long-term evaluation of BM-MDSC tracking and fate, it may be useful to develop alternative methods of cell labeling. Other approaches include radioactive cell labeling using [^64^Cu]PTSM or ^64^Cu-labeled monoclonal antibodies for quantitative positron emission tomography (PET) and iron oxide labeling, or ^19^F-labeling for magnetic resonance imaging (MRI) [45–47]. Combined PET/MRI is superior to OI as it enables holistic three-dimensional whole body tracking, which would allow visualization of BM-MDSCs for longer time periods and at higher resolution. Co-localization of BM-MDSCs within different tumor regions, e.g. necrotic centers, areas of angiogenesis, invasive growth areas, is possible using adequate imaging technology and would enable the detailed characterization of the homing patterns of BM-MDSCs. Moving forward, it would be interesting to isolate the M-MDSC-like fraction of BM-MDSCs (CD11b/Ly6C^hi^/Ly6G^-^) and compare and contrast their homing, localization, plasticity and differentiation in breast tumor-bearing mice and other cancer types. Furthermore, very little is known regarding the involvement of MDSCs in metastatic tumor microenvironments and for example, in the formation of pre-metastatic niches, in which they have been reported to accumulate and promote metastatic breast tumor outgrowth [48]. Determining how MDSC differentiation and function is affected within different tumor microenvironments may also help to identify novel methods of therapeutic intervention, thereby enabling targeting of MDSCs to reduce immunosuppression.

## Supporting information

S1 Table Primer sequences for RT-PCR. (DOCX)

S2 Table Monoclonal anti-mouse antibody details used for flow cytometry. (DOCX)

S1 Fig Quantification of DiD-BM-MDSC accumulation in *ex vivo* organs. **A)** Quantification of radiant efficiency of FL-signal for *ex vivo* lung, liver and spleen 7 days after i.v., i.c. or i.p. injection of 1x10^6^ DiD-labeled BM-MDSCs into naïve mice as shown in Fig 2E. **B)** Quantification of radiant efficiency of FL-signal for *ex vivo* lung, liver, spleen and tumor from Luc-PyMT tumor-bearing mice on day 21; 7 days after DiD-BM-MDSCs injection from Fig 3D. Data represented as mean ± SEM; n = 3–5 mice for all groups.
(TIF)

S2 Fig DiD-BM-MDSCs do not home to lungs or liver in mice with established metastases. **A-B)** Representative *ex vivo* images of DiD-BM-MDSC (FL signal; top panel) localization to liver (A) and lung (B) 2 weeks after i.v. injection of DiD-BM-MDSCs into mice with metastatic tumors (BL signal; bottom panel) from Fig 4. Quantification of radiant efficiency (RE) of FL-signal shown on the right. Naïve C57Bl/6 mice were used as controls. Data represented as mean ± SEM; *p<0.05; ***p<0.001; n = 3–5 mice for all groups. RE = Radiant Efficiency; R = Radiance.
(TIF)

S3 Fig Adoptive transfer of BM-MDSCs does not alter primary tumor growth. **A)** Schematic of treatment regimen for survival analysis after adoptive transfer of BM-MDSCs into tumor-bearing mice. Mice were orthotopically injected with 5x10^5^ PyMT-WT cells into the MFP on day 0, and i.v. injected with 1x10^6^ or 4x10^6^ BM-MDSCs on day 10 and 20. Primary tumor growth was monitored to endstage. **B,C**) Individual tumor growth curves (**B**) and Kaplan-Meier survival curves (**C**) after i.v. injection of 1x10^6^ BM-MDSCs at day 10 and 20 (primary tumor n = 5; primary tumor + BM-MDSCs n = 10). **D,E**) Individual tumor growth curves (**D**) and Kaplan-Meier survival curve (**E**) for mice injected with 4x10^6^ BM-MDSCs on day 10 and day 20 (primary tumor alone n = 5; primary tumor + BM-MDSCs n = 6).
(TIF)

S4 Fig Co-injection of BM-MDSCs does not alter primary tumor growth. **A,B**) Individual tumor growth curves (**A**) and Kaplan-Meier survival curve (**B**) for mice injected with 5x10^5^ PyMT-WT tumor cells alone (primary tumor n = 5) or co-injected with 5x10^5^ BM-MDSCs in the mammary fat pad (primary tumor + BM-MDSCs n = 7).
(TIF)

S5 Fig Tumor microenvironment dynamics after adoptive transfer of BM-MDSCs. **A**) Schematic of treatment regimen for tumor microenvironment analysis after adoptive transfer of DiD-BM-MDSCs into tumor-bearing mice. Mice were orthotopically injected with 5x10^5^ PyMT-WT cells into the MFP on day 0, and i.v. injected with 1x10^6^ DiD-BM-MDSCs on day 10 and 20. Tumors and organs were harvested and analyzed by flow cytometry at 48 hours (day 22) or 7 days (day 27) after the second BM-MDSC injection. Tumors from mice injected with PyMT-WT cells alone were used as controls. **B-D**) Flow cytometry analysis of CD3 lymphocyte populations (**B**) as a percentage of CD45.2, Ly6C and Ly6G myeloid cell populations (**C**) as a percentage of CD45.2/CD11b cells, and macrophage and dendritic cell populations (**D**) as a percentage of CD45.2/MHC Class II cells within the primary tumor 48 hours (n = 4 per group) and 7 days (n = 6 per group) after second BM-MDSC injection. Data represented as mean ± SEM. **p<0.01; ***p<0.001.
(TIF)

S6 Fig Microenvironment dynamics in the spleen and lungs after adoptive transfer of BM-MDSCs into breast tumor-bearing mice. (**A-C**) Flow cytometry analysis of CD3 lymphocyte (**A**), CD11b myeloid (**B**) and macrophage and dendritic cell (**C**) populations within the spleen of mice injected with 1x10^6^ BM-MDSCs i.v. on day 10 and 20 after PyMT-WT tumor cell injection (n = 4 for all groups). Spleens were analyzed 48 hours (day 22) and 5 days (day 25) after second injection of BM-MDSCs. Spleens from PyMT-WT tumor-bearing mice were used as controls. (**D-F**) Flow cytometry analysis of CD3 lymphocytes (**D**), CD11b myeloid (**E**) and macrophage and dendritic populations (**F**) in the lungs of mice injected with 1x10^6^ BM-MDSCs i.v. on day 10 and 20 after PyMT-WT tumor cell injection (n = 4 for all groups). Lungs were analyzed 48 hours (day 22) and 5 days (day 25) after second injection of BM-MDSCs. Lungs from PyMT-WT tumor-bearing mice were used as controls. Data represented as mean ± SEM. *p<0.05; **p<0.01; ***p<0.001.
(TIF)

S7 Fig Spleen microenvironment dynamics after adoptive transfer of DiD-BM-MDSCs into tumor-bearing mice. **A**) Flow cytometry analysis of CD3 lymphocytes, CD3/CD4 and CD3/CD8 T cells, as well as CD3^-^/NK1.1^+^ NK cells from the spleen of tumor-bearing mice (control) and after adoptive transfer of DiD-BM-MDSCs at day 24 (48 hours after second dose of DiD-BM-MDSCs). **B**) Flow cytometry analysis of the percentage of Foxp3^+^ cells within the CD3/CD4 T cell population in the spleen at day 24. **C**) Flow cytometry analysis of CD11b/Ly6C myeloid populations as a percentage of CD45.2^+^ cells within the spleen at day 24. **D**) Flow cytometry analysis of DCs and macrophages as a percentage of CD45.2^+^ cells within the spleen at day 24. **E**) Representative flow cytometry plots of CD11b/Ly6C populations within the spleen at day 24. For all analyses, control n = 5; DiD-BM-MDSC n = 6. Data represented as mean ± SEM. *p<0.05; n.s. not significant.
(TIF)

## Abbreviations

Arg1: Arginase 1
BL: Bioluminescence
BMDCs: Bone marrow-derived cells
BM-MDSCs: Bone Marrow myeloid-derived suppressor cells
DCs: Dendritic cells
FL: Fluorescence
FACS: Fluorescence activated cell sorting
i.c.: Intra cardiac
iNos: Inducible nitric oxide synthase
IL-6: Interleukin 6
i.p.: Intra peritoneal
i.v.: Intra venous
GM-CSF: Granulocyte-macrophage colony-stimulating factor
G-MDSCs: Granulocytic myeloid-derived suppressor cells
Luc: Luciferase
MRI: Magnetic Resonance Imaging
MFP: Mammary fat pad
MDSCs: Myeloid-derived suppressor cells
M-MDSCs: Monocytic myeloid-derived suppressor cells
NK cells: Natural killer cells
OI: Optical imaging
PET: Positron Emission Tomography
PyMT-MMTV: Polyoma middle-T mouse mammary tumor virus
Tregs: Regulatory T helper cells
TME: Tumor microenvironment
