# Supplementary material for: Tracking the fate of adoptively transferred myeloid-derived suppressor cells in the primary breast tumor microenvironment
Source: PLoS One. 2018 Apr 20;13(4):e0196040. doi: 10.1371/journal.pone.0196040 (PMC5909918; doi:10.1371/journal.pone.0196040)
Supplement: S1 Table — (DOCX) [file pone.0196040.s001.docx]

**S1 Table. Primer sequences for RT-PCR.**

| **Primer** | **Sense** | **Antisense** | **GenBank Accession Number** |
| --- | --- | --- | --- |
| S100A8 | 5ʹ-TGAGCAACCTC ATTGATGTCTACC-3ʹ | 5'-ATGCCACACC CACTTTTATCACC-3ʹ | [NM_013650.2](https://www.ncbi.nlm.nih.gov/nucleotide/NM_013650.2?report=genbank&log$=nucltop&blast_rank=1&RID=BX61SHPW015) |
| S100A9 | 5’-GAAGAAAGAGAA GAGAAATGAAGCC-3ʹ | 5'- CTTTGCCATCAGC ATCATACACTCC-3ʹ | NM_009114.3 |
| Nos3 | 5’-CTCCAGCACCGGAGCCTA-3’ | 5’-TACAGGGCCCATC CTGCT-3’ | NM_008713.4 |
| Arg1 | 5‘-ACAAGACAGGGC TCCTTTCAG-3‘ | 5‘-GGCTTATGGTTAC CCTCCCG-3‘ | NM_007482.3 |
| Arg2 | 5’-AATCCCCTCCCTG CCAATCA-3’ | 5‘-CACTCCTAGCTTC TTCTGTCCC-3‘ | NM_009705.3 |
| Aldolase | 5′-TGGGCCTTGACTT TCTCCTAT-3′ | 5′-TGTTGATGGAGCA GCCTTAGT-3′ | NM_001177307.1 |
