# Supplementary material for: Tracking the fate of adoptively transferred myeloid-derived suppressor cells in the primary breast tumor microenvironment
Source: PLoS One. 2018 Apr 20;13(4):e0196040. doi: 10.1371/journal.pone.0196040 (PMC5909918; doi:10.1371/journal.pone.0196040)
Supplement: S2 Table — (DOCX) [file pone.0196040.s002.docx]

**S2 Table. Monoclonal anti-mouse antibody details used for flow cytometry.**

| **Antibody** | **Clone** | **Product Number** | **Company** |
| --- | --- | --- | --- |
| CD45.2 | 104 | 47-0454-80 | eBioscience |
| CD11b | M1/70 | 48-0112-80 | eBioscience |
| CD3ε | 145-2C11 | 11-0031-81 | eBioscience |
| NK1.1 | PK136 | 25-5941-81 | eBioscience |
| CD4 | RM4-5 | 563106 | BD Horizon |
| CD8α | 53-6.7 | 553033 | BD Bioscience |
| F4/80 | BM8 | 12-4801-80 | eBioscience |
| MHC Class II | M5/114.15.2 | 48-5321-80 | eBioscience |
| CD11c | N418 | 17-0114-81 | eBioscience |
| CD62L | MEL-14 | 564108 | BD Horizon |
| CD80 | 16-10A1 | 12-0801-82 | eBioscience |
| CD86 | GL-1 | 11-0862-82 | eBioscience |
| CD115 | AFS98 | 25-1152-80 | BD Pharmingen |
| Ly6G | 1A8 | 561104 | BD Horizon |
| Ly6C | AL-21 | 560595 | BD Horizon |
