# Supplementary figures and images for: Tracking the fate of adoptively transferred myeloid-derived suppressor cells in the primary breast tumor microenvironment
Source: PLoS One. 2018 Apr 20;13(4):e0196040. doi: 10.1371/journal.pone.0196040 (PMC5909918; doi:10.1371/journal.pone.0196040)

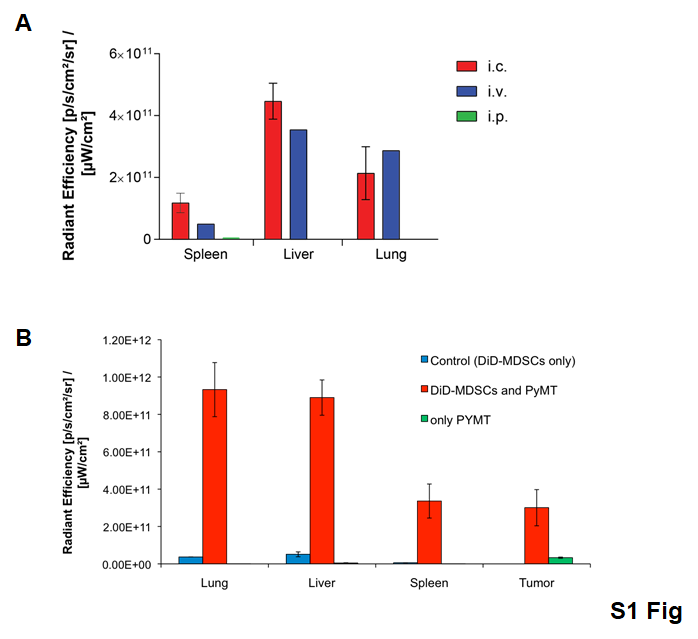

Supplement: S1 Fig — A) Quantification of radiant efficiency of FL-signal for ex vivo lung, liver and spleen 7 days after i.v., i.c. or i.p. injection of 1x10^6^ DiD-labeled BM-MDSCs into naïve mice as shown in Fig 2E. B) Quantification of radiant efficiency of FL-signal for ex vivo lung, liver, spleen and tumor from Luc-PyMT tumor-bearing mice on day 21; 7 days after DiD-BM-MDSCs injection from Fig 3D. Data represented as mean ± SEM; n = 3–5 mice for all groups. (TIF) [file pone.0196040.s003.tif]

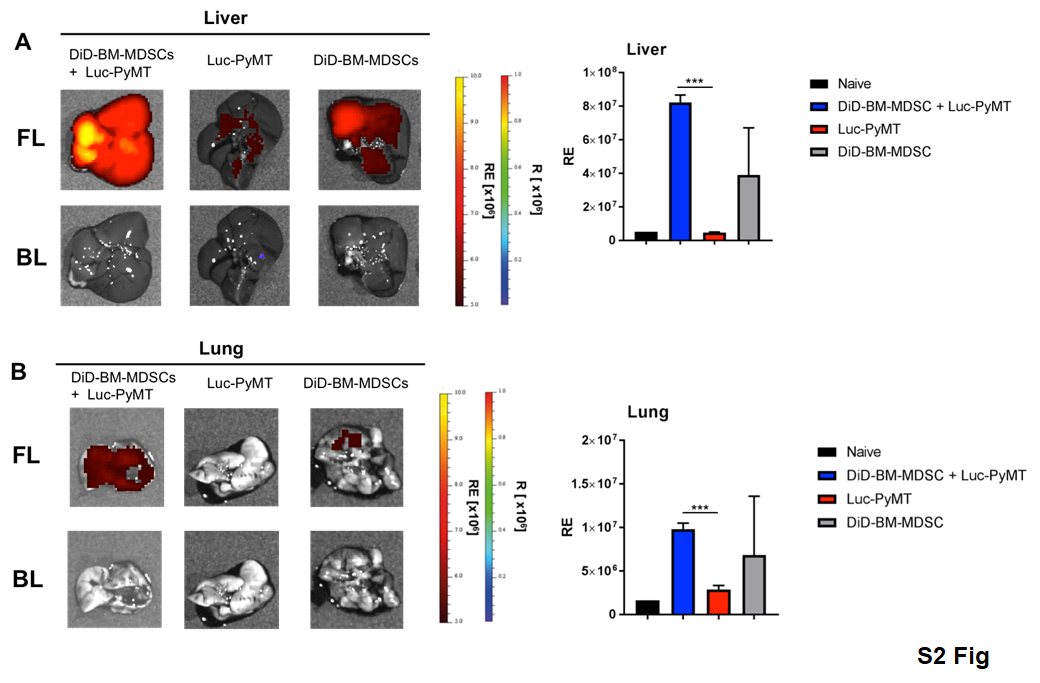

Supplement: S2 Fig — A-B) Representative ex vivo images of DiD-BM-MDSC (FL signal; top panel) localization to liver (A) and lung (B) 2 weeks after i.v. injection of DiD-BM-MDSCs into mice with metastatic tumors (BL signal; bottom panel) from Fig 4. Quantification of radiant efficiency (RE) of FL-signal shown on the right. Naïve C57Bl/6 mice were used as controls. Data represented as mean ± SEM; *p<0.05; ***p<0.001; n = 3–5 mice for all groups. RE = Radiant Efficiency; R = Radiance. (TIF) [file pone.0196040.s004.tif]

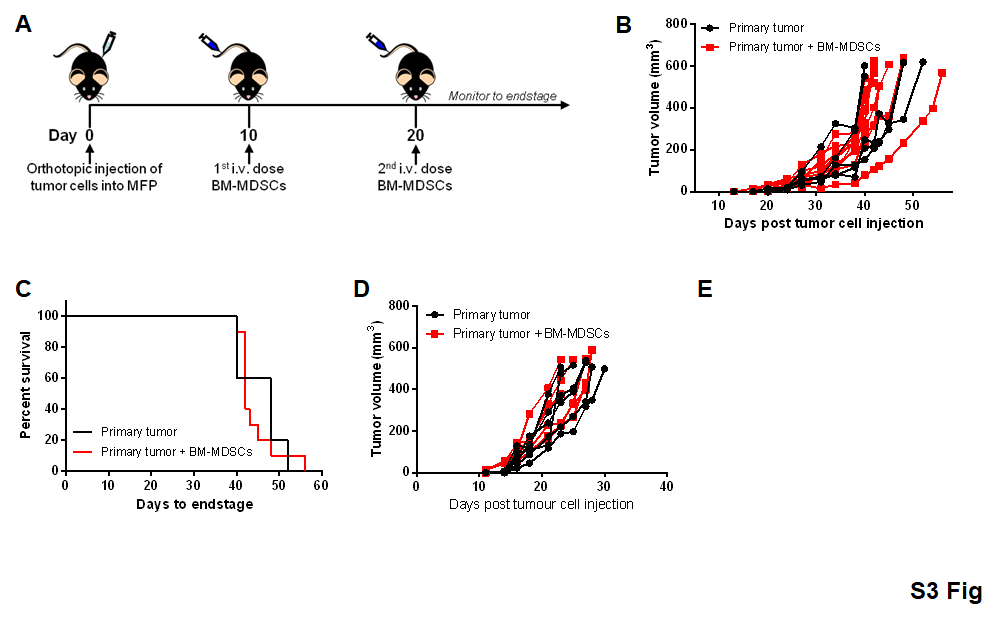

Supplement: S3 Fig — A) Schematic of treatment regimen for survival analysis after adoptive transfer of BM-MDSCs into tumor-bearing mice. Mice were orthotopically injected with 5x10^5^ PyMT-WT cells into the MFP on day 0, and i.v. injected with 1x10^6^ or 4x10^6^ BM-MDSCs on day 10 and 20. Primary tumor growth was monitored to endstage. B,C) Individual tumor growth curves (B) and Kaplan-Meier survival curves (C) after i.v. injection of 1x10^6^ BM-MDSCs at day 10 and 20 (primary tumor n = 5; primary tumor + BM-MDSCs n = 10). D,E) Individual tumor growth curves (D) and Kaplan-Meier survival curve (E) for mice injected with 4x10^6^ BM-MDSCs on day 10 and day 20 (primary tumor alone n = 5; primary tumor + BM-MDSCs n = 6). (TIF) [file pone.0196040.s005.tif]

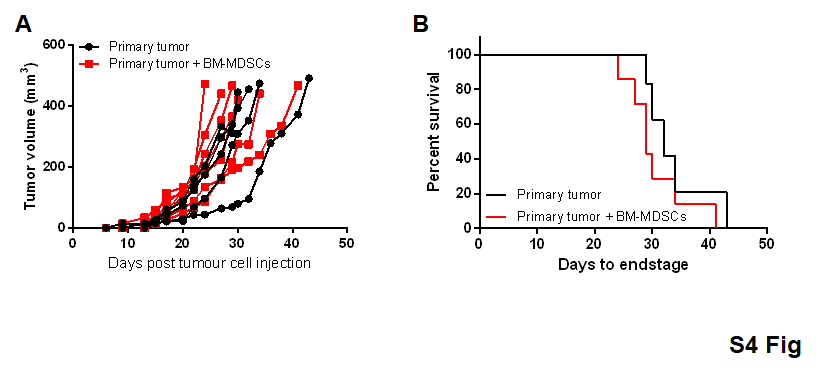

Supplement: S4 Fig — A,B) Individual tumor growth curves (A) and Kaplan-Meier survival curve (B) for mice injected with 5x10^5^ PyMT-WT tumor cells alone (primary tumor n = 5) or co-injected with 5x10^5^ BM-MDSCs in the mammary fat pad (primary tumor + BM-MDSCs n = 7). (TIF) [file pone.0196040.s006.tif]

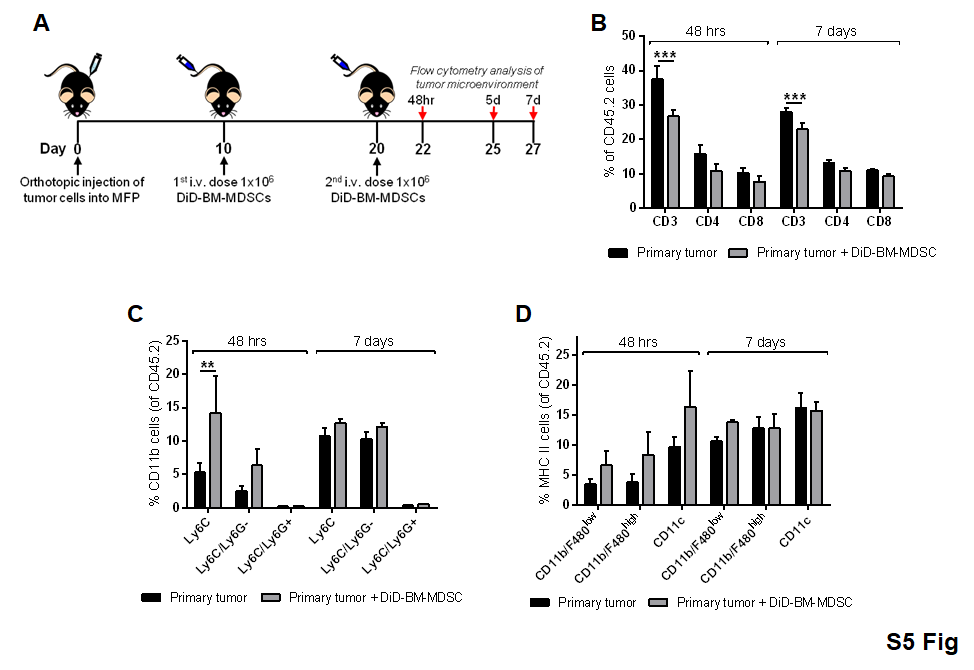

Supplement: S5 Fig — A) Schematic of treatment regimen for tumor microenvironment analysis after adoptive transfer of DiD-BM-MDSCs into tumor-bearing mice. Mice were orthotopically injected with 5x10^5^ PyMT-WT cells into the MFP on day 0, and i.v. injected with 1x10^6^ DiD-BM-MDSCs on day 10 and 20. Tumors and organs were harvested and analyzed by flow cytometry at 48 hours (day 22) or 7 days (day 27) after the second BM-MDSC injection. Tumors from mice injected with PyMT-WT cells alone were used as controls. B-D) Flow cytometry analysis of CD3 lymphocyte populations (B) as a percentage of CD45.2, Ly6C and Ly6G myeloid cell populations (C) as a percentage of CD45.2/CD11b cells, and macrophage and dendritic cell populations (D) as a percentage of CD45.2/MHC Class II cells within the primary tumor 48 hours (n = 4 per group) and 7 days (n = 6 per group) after second BM-MDSC injection. Data represented as mean ± SEM. **p<0.01; ***p<0.001. (TIF) [file pone.0196040.s007.tif]

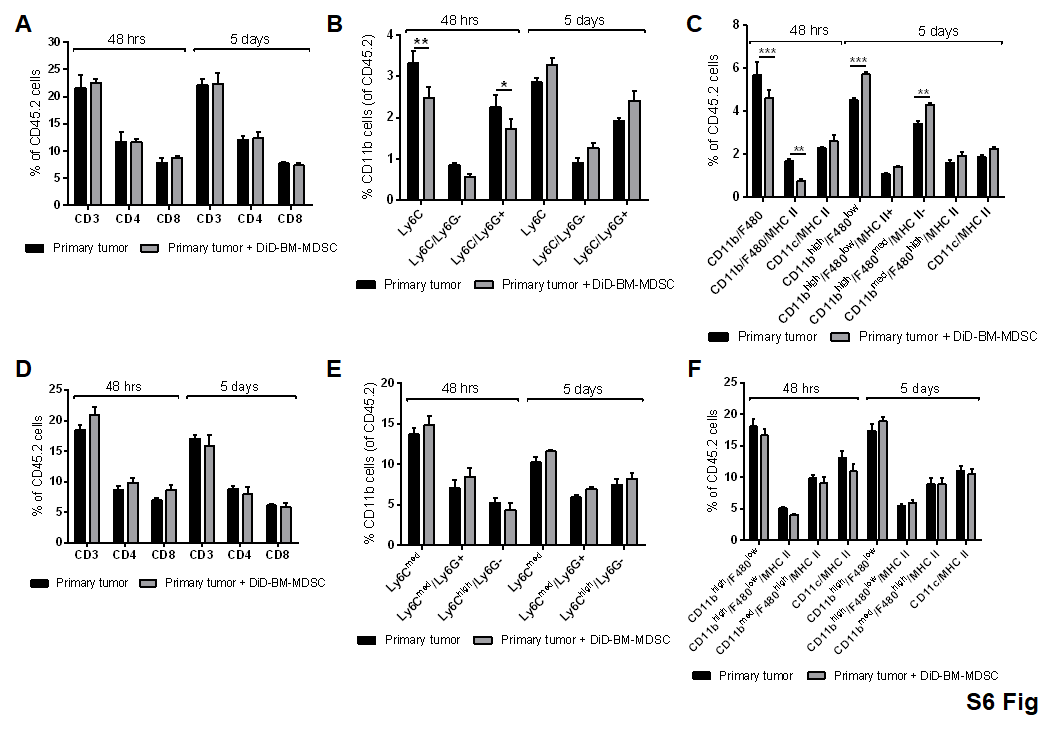

Supplement: S6 Fig — (A-C) Flow cytometry analysis of CD3 lymphocyte (A), CD11b myeloid (B) and macrophage and dendritic cell (C) populations within the spleen of mice injected with 1x10^6^ BM-MDSCs i.v. on day 10 and 20 after PyMT-WT tumor cell injection (n = 4 for all groups). Spleens were analyzed 48 hours (day 22) and 5 days (day 25) after second injection of BM-MDSCs. Spleens from PyMT-WT tumor-bearing mice were used as controls. (D-F) Flow cytometry analysis of CD3 lymphocytes (D), CD11b myeloid (E) and macrophage and dendritic populations (F) in the lungs of mice injected with 1x10^6^ BM-MDSCs i.v. on day 10 and 20 after PyMT-WT tumor cell injection (n = 4 for all groups). Lungs were analyzed 48 hours (day 22) and 5 days (day 25) after second injection of BM-MDSCs. Lungs from PyMT-WT tumor-bearing mice were used as controls. Data represented as mean ± SEM. *p<0.05; **p<0.01; ***p<0.001. (TIF) [file pone.0196040.s008.tif]

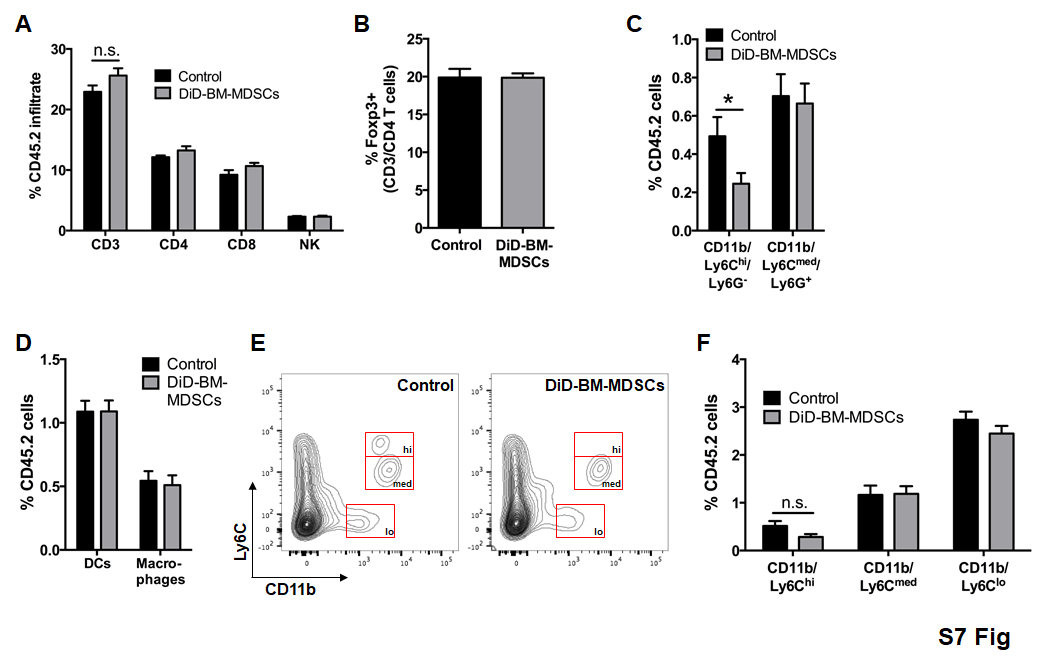

Supplement: S7 Fig — A) Flow cytometry analysis of CD3 lymphocytes, CD3/CD4 and CD3/CD8 T cells, as well as CD3-/NK1.1+ NK cells from the spleen of tumor-bearing mice (control) and after adoptive transfer of DiD-BM-MDSCs at day 24 (48 hours after second dose of DiD-BM-MDSCs). B) Flow cytometry analysis of the percentage of Foxp3+ cells within the CD3/CD4 T cell population in the spleen at day 24. C) Flow cytometry analysis of CD11b/Ly6C myeloid populations as a percentage of CD45.2+ cells within the spleen at day 24. D) Flow cytometry analysis of DCs and macrophages as a percentage of CD45.2+ cells within the spleen at day 24. E) Representative flow cytometry plots of CD11b/Ly6C populations within the spleen at day 24. For all analyses, control n = 5; DiD-BM-MDSC n = 6. Data represented as mean ± SEM. *p<0.05; n.s. not significant. (TIF) [file pone.0196040.s009.tif]
